# Engineering *Escherichia coli* for Isobutanol Production from Xylose or Glucose–Xylose Mixture

**DOI:** 10.3390/microorganisms11102573

**Published:** 2023-10-16

**Authors:** Pengfei Gu, Fangfang Li, Zhaosong Huang

**Affiliations:** 1 School of Biological Science and Technology, University of Jinan, Jinan 250022, China; bio_huangzs@ujn.edu.cn; 2 Yantai Food and Drug Control and Test Center, Yantai 264003, China; loveecoli@163.com

**Keywords:** Dahms pathway, Ehrlich pathway, Entner–Doudorof pathway, isobutanol, metabolic engineering, xylose, glucose

## Abstract

Aiming to overcome the depletion of fossil fuels and serious environmental pollution, biofuels such as isobutanol have garnered increased attention. Among different synthesis methods, the microbial fermentation of isobutanol from raw substrate is a promising strategy due to its low cost and environmentally friendly and optically pure products. As an important component of lignocellulosics and the second most common sugar in nature, xylose has become a promising renewable resource for microbial production. However, bottlenecks in xylose utilization limit its wide application as substrates. In this work, an isobutanol synthetic pathway from xylose was first constructed in *E. coli* MG1655 through the combination of the Ehrlich and Dahms pathways. The engineering of xylose transport and electron transport chain complexes further improved xylose assimilation and isobutanol production. By optimizing xylose supplement concentration, the recombinant *E. coli* strain BWL4 could produce 485.35 mg/L isobutanol from 20 g/L of xylose. To our knowledge, this is the first report related to isobutanol production using xylose as a sole carbon source in *E. coli*. Additionally, a glucose–xylose mixture was utilized as the carbon source. The Entner–Doudorof pathway was used to assimilate glucose, and the Ehrlich pathway was applied for isobutanol production. After carefully engineering the recombinant *E. coli*, strain BWL9 could produce 528.72 mg/L isobutanol from a mixture of 20 g/L glucose and 10 g/L xylose. The engineering strategies applied in this work provide a useful reference for the microbial production of isobutanol from xylose or glucose–xylose mixture.

## 1. Introduction

As an important platform chemical compound for butyl rubber, lubricant, and polyester synthesis, isobutanol has been widely applied in the fields of foods, pharmaceuticals, chemicals, and so on [1]. In addition, when compared with traditional biofuels, such as ethanol, isobutanol has a higher energy density, higher octane number, and lower hygroscopicity [2,3]. Accordingly, isobutanol has become a very promising biofuel and has attracted more attention recently. Leading up to now, many efforts have been made investigating how to obtain pure isobutanol at an industrial scale, which has been mainly dependent upon chemical synthesis or microbial fermentation [4]. With the advantages of an environmentally friendly fermentation process, the utilization of cheap raw substrates, mild reaction conditions, and pure production in optical activity, the microbial production of isobutanol has become a promising synthetic strategy for isobutanol [5]. Thus, engineering an excellent microbial chassis for isobutanol production is desirable for expanding the application of isobutanol.

Several different carbon sources, such as glucose [6,7,8], cellobiose [9], cellobionic acid [10], acetate [11] and cheese whey [12], have been explored as substrates for microbial isobutanol production. As widely used industrial organisms, such as *Escherichia coli*, do not have the native capacity for isobutanol synthesis, the construction of an effective isobutanol biosynthetic pathway was the first obstacle to overcome. By combining a branched-chain amino acid synthetic pathway and an Ehrlich pathway, a synthetic pathway for isobutanol production from glucose was constructed previously [13]. In this pathway, pyruvate derived from a glycolytic pathway was converted into 2-ketoisovalerate through acetolactate synthase AlsS from *Bacillus subtilis*, and keto-acid reductoisomerase IlvC and dihydroxy-acid dehydratase IlvD from *E. coli*, in turn. Then, 2-ketoisovalerate was transformed into isobutyraldehyde by 2-ketoisovalerate decarboxylase, which was encoded by *kivD* from *Lactococus lactis* IL1403. Finally, alcohol dehydrogenase AdhA from *L. lactis*, or YqhD from *E. coli*, could achieve isobutanol production from isobutyraldehyde. By using this typical pathway combined with inactivation of competing pathways, about 22 g/L of isobutanol was obtained from recombinant *E. coli* under a micro-aerobic condition [13]. On this basis, the in situ product removal strategy was employed to release the toxicity of isobutanol for the host, and isobutanol production was increased by a large margin to 50.8 g/L [14].

Apart from glucose, other carbon sources have also been investigated for the production of isobutanol. For example, cheese whey is a byproduct of cheese’s production from milk, and contains lactose, protein, fat, lactate, calcium, phosphate, and chloride. Accordingly, it can be employed as a potential substrate for isobutanol production in *E. coli*. Consistent with expectations, raw cheese whey can support the normal growth of recombinant *E. coli* WΔ*ldhA*Δ*adhE*Δ*pta*Δ*frdA* IB4, and 19.6 g/L of isobutanol can be accumulated for this strain [12]. 

Biomass, such as non-food lignocellulosics, represents a class of abundant resources which is still not being sufficiently developed [15]. Xylose derived from lignocellulosics is the second-most-common sugar in nature and xylose accounts for 18–30% of lignocellulose hydrolysate sugars [16]. Therefore, xylose has become a promising renewable resource for producing biofuels and chemicals. However, due to bottlenecks in the xylose metabolism, only a few products are synthesized from xylose in contrast to glucose. Accordingly, improving xylose utilization efficiency in recombinant microorganisms is vital for the valuable production of chemicals from xylose or even lignocellulosics. 

The utilization of xylose in microorganisms is mainly accomplished using the isomerase pathway (XIP) or the Oxo-reductive pathway (ORP) [17,18]. In the XIP, D-xylose is first isomerized into D-xylulose, and then D-xylulose is phosphorylated into D-xylulose-5-phosphate. Finally, D-xylulose-5-phosphate is employed to synthesize cellular metabolites through the pentose phosphate pathway (PPP). Comparatively, D-xylose is first reduced to D-xylitol in the ORP, and then is transformed into D-xylulose. Similar to XIP, D-xylulose is phosphorylated and also enters into the PPP. Notably, the ORP pathway can also be referred to as the XR-XDH pathway. Apart from these two common pathways, the xylose oxidative pathway (XOP) in some bacteria and archaea exhibits an alternative pathway for xylose metabolism. In the XOP pathway, xylose is first transformed into D-xylonolactone by xylose dehydrogenase. D-xylonolactone is then hydrolyzed either spontaneously or by lactonases to form D-xylonic acid. Afterwards, 2-keto-3-deoxy-D-xylonic acid (KDX) is generated from D-xylonic acid by xylonate dehydratase. KDX can be further metabolized through one of two separate routes: the Weimberg pathway or the Dahms pathway. In the Weimberg pathway, KDX is transformed into α-ketoglutarate, which is an important intermediate for the tricarboxylic acid cycle. Alternatively, in the Dahms pathway, KDX is converted into pyruvate and glycolaldehyde by an aldolase, and both compounds can be assimilated for cellular metabolism. 

Considering *E. coli* is a widely applied microbial chassis for the production of valuable chemicals, and has the advantages of its convenient genetic engineering and its fast growth in cheap media, it was employed for isobutanol production in this work. As xylose represents a cheap substrate and isobutanol is a promising, advanced biofuel, we aimed to produce isobutanol from xylose in *E. coli* for the first time. The xylose assimilation and Ehrlich pathways were first combined and constructed into an entire isobutanol synthetic pathway from xylose. Considering pyruvate is the direct precursor of isobutanol, the Dahms pathway was selected for xylose utilization. By engineering a xylose transport system, an electron transport chain complex, and a xylose supplement concentration, strain BWL4 could produce 485.35 mg/L of isobutanol from xylose. To further increase the isobutanol titer, the *E. coli* strain was re-engineered and the Entner–Doudorof pathway was employed for glucose assimilation. By using glucose and xylose as mixed carbon sources, the isobutanol production of recombinant *E. coli* BWL9 was further improved.

## 2. Materials and Methods

### 2.1. Bacterial Strains 

All strains, plasmids, and primers used in this study are listed in Table 1, Table 2, and Table S1, separately. Wild *E. coli* BW25113 was used in constructing different isobutanol-producing strains. Wild *E. coli* DH5α was devoted to vector construction. 

### 2.2. Plasmid Construction

To facilitate the construction process, recombinant plasmid pLL1 containing *xylD*(Cc) that encodes xylose dehydrogenase from *C. crescentus*, *yagF* that encodes xylonate dehydratase, *yagE* that encodes 2-dehydro-3-deoxy-D-pentonate aldolase, *aldA* encoding lactaldehyde dehydrogenase, and *aceAK* that encodes isocitrate lyase-isocitrate dehydrogenase kinase/phosphatase was directly synthesized by TSINGKE Biological Technology. Additionally, pLL2 containing *adhA*(Ll) that encodes alcohol dehydrogenase from *L. lactis*, *kivD*(Ll) that encodes 2-keto acid decarboxylase from *L. lactis*, *ilvC* that encodes ketol-acid reductoisomerase, *ilvD* that encodes dihydroxy-acid dehydratase, and *alaS*(Bs) that encodes acetolactate synthase from *B. subtilis* was also directly synthesized by TSINGKE Biological Technology.

### 2.3. Gene Deletion

Three genes, *ldhA*, *pflB,* and *xylAB,* in *E. coli* BW25113, which encode lactate dehydrogenase, pyruvate-formate lyase, and xylulokinase, respectively, were knocked out in BW25113 sequentially using a one-step inactivation method [19]. The primers ldhA-QF/ldhA-QR, pflB-QF/pflB-QR, and xylAB-QF/xylAB-QR, along with the template plasmids pKD3 or pKD4, were used for obtaining targeting DNA fragments of *ldhA*, *pflB,* and *xylAB*, respectively. Positive clones were verified via PCR using ldhA-JF/ldhA-JR, pflB-JF/pflB-JR, and xylAB-JF/xylAB-JR. The resulting strain of BW25113 (Δ*ldhA*Δ*pflB*Δ*xylAB*) was titled BWL1. Based on BWL1, the *cbdAB* gene encoding terminal oxidases was also deleted utilizing the same strategy. To replace *cyo* encoding cytochrome O with the *xdh* that encodes xylose dehydrogenase from *Caulobacter crescentus* in BWL1, DNA fragments containing homologous arms of the *cyo*, *xdh* gene and the chloramphenicol resistant gene *cmr* were obtained using overlap PCR. In brief, the upstream homologous arm of *cyo* and *cmr* was assembled via the primers cyo-cmr-NF/cmr-NR and template plasmid pKD3, while the downstream homologous arm of *cyo* and *xdh* genes was assembled using the genome of *C. crescentus* as a template along with primers cmr-xdh-NF/xdh-cyo-NR. Then, these two DNA fragments were further assembled using overlap PCR. The homologous arms of *cyo* were further extended using the primers cyo-cmr-NF plus/xdh-cyo-NR plus. The replacement of *cyo* was performed according to the one-step inactivation method [19]. Positive clones were verified with primers cyo-JF/cyo-JR. Similarly, *nuo* encoding NADH-quinone oxidoreductase was replaced by the *xylFGH* encoding xylose ABC transporter periplasmic binding protein. The resulting strain was referred to as BWL3. Finally, pLL1 and pLL2 plasmids were co-transformed into BWL1 and BWL3 to obtain BWL2 and BWL4, respectively.

To construct recombinant *E. coli* using a glucose–xylose mixture as the carbon source, BWL0 was selected as the base strain. The *ptsG* gene was first knocked out in BWL0 via the one-step inactivation method [19]. Subsequently, *pgi* encoding glucose-6-phosphate isomerase was replaced by *zwf* encoding glucose-6-phosphate 1-dehydrogenase, similar to the replacement of *cyo* via the one-step inactivation method [19]. The resulting strain was referred to as BWL6. Afterwards, *gnd* encoding 6-phosphogluconate dehydrogenase, *pta* encoding phosphate acetyltransferase, and *ackA* encoding acetate kinase were replaced with *pgl* encoding 6-phosphogluconolactonase, *edd* encoding phosphogluconate dehydratase, and *eda* encoding 2-dehydro-3-deoxyphosphogluconate aldolase, respectively, in BWL6 via the same method; in addition, the recombinant *E. coli* BWL7, BWL8, and BWL9 were obtained, in turn. 

### 2.4. Growth Conditions

Luria–Bertani medium (1% tryptone, 0.5% yeast extract, and 1% NaCl) was used for culturing *E. coli* strains at 37 °C for 8–12 h. Different antibiotics were supplemented with appropriate concentrations, including ampicillin (100 mg/L), chloramphenicol (17 mg/L), kanamycin (25 mg/L), and spectinomycin (50 mg/L). For batch fermentation, a medium containing 33.9 g/L Na_2_HPO_4_, 15 g/L KH_2_PO_4_, 2.5 g/L NaCl, 5 g/L NH_4_Cl, 1 mM MgSO_4_, 0.1 mM CaCl_2_, 5 g/L yeast extract, and 10 g/L xylose was employed. Glucose was supplemented as indicated. An amount of 1 mL of overnight cells were inoculated into 50 mL fermentation medium for batch fermentation, and strains were cultivated at 37 °C with 200 rpm of shaking. Isopropyl β-D-1-thiogalactopyranoside (IPTG) was added at a final concentration of 0.2 mM, when the OD_600_ of *E. coli* cells reached 0.4–0.6. 

### 2.5. Analytical Methods

High-performance liquid chromatography (Thermo Fisher Scientific, Waltham, MA, USA) and Aminex HPX-87H ion exclusion particles (300 mm × 7.8 mm, Bio-Rad, Hercules, CA, USA) were used for determining the concentrations of glucose, xylose, and isobutanol, respectively. The mobile phase was 5 mM sulfuric acid with a flow rate of 0.6 mL/min, and the column was maintained at 65 °C. For glucose determination, a refractive index detector was applied with a 10 μL sample. For isobutanol determination, a UV detector was employed at 214 nm with a 10 μL sample. Cell growth was monitored via OD_600_ using a UV5100H spectrophotometer (METASH, Shanghai, China). Three parallel experiments were carried out for each experiment within this work. The error bars represent standard deviations from three replicate experiments. The Origin 2019 and Microsoft Excel 2019 programs were employed for statistical analyses. Data were plotted using Origin 2019.

## 3. Results and Discussion 

### 3.1. Construction of an Isobutanol Synthetic Pathway from Xylose

**Figure 1:**
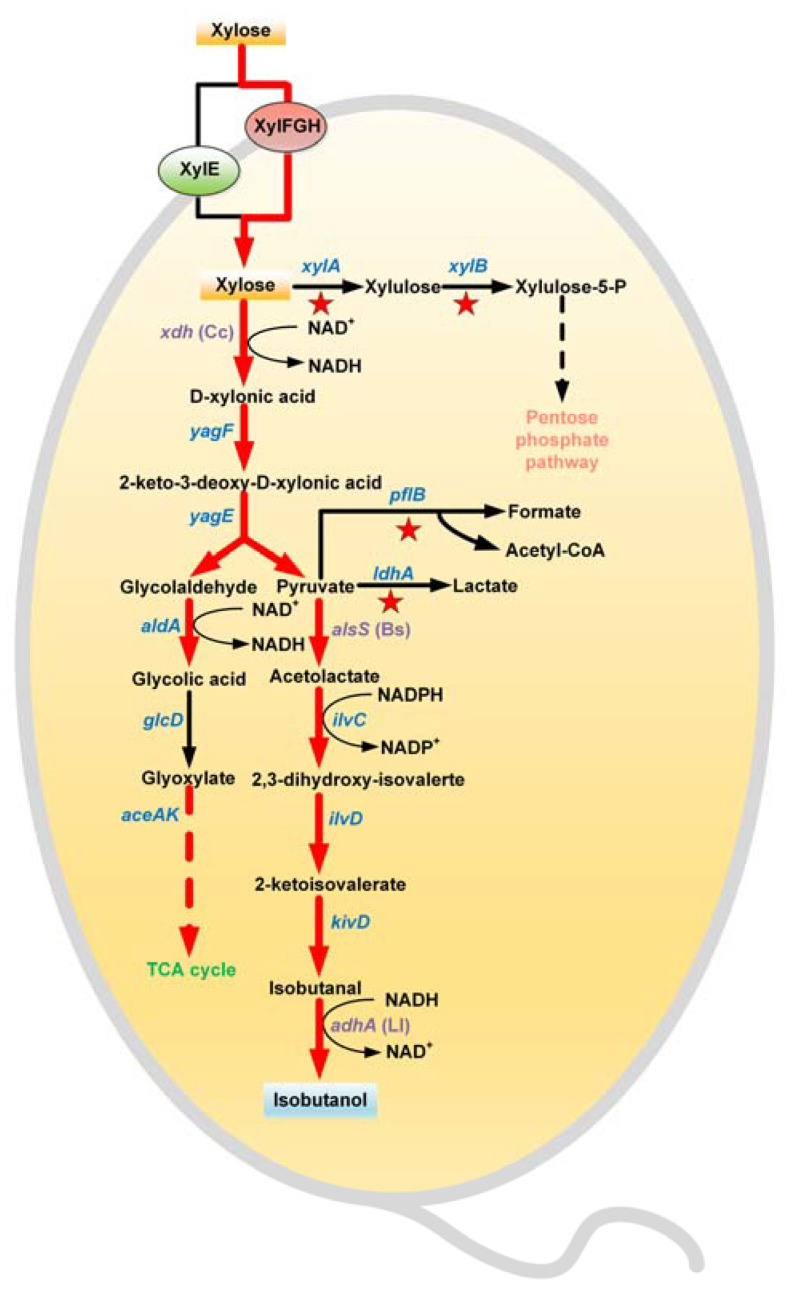
Isobutanol synthetic pathway from xylose in *E. coli*. The red, five-pointed stars indicate the genes that were deleted. The thick red arrows indicate the increased flux by directly overexpressing the corresponding genes within the plasmids. Heterogenous genes are indicated in purple.

As isobutanol is not a natural product of wild *E. coli*, a complete synthetic pathway for isobutanol from xylose should be constructed first. In this study, the entire pathway could be divided into two parts: part I was located from substrate xylose to an intermediate pyruvate, and part 2 was located from pyruvate to end-product isobutanol (Figure 1). To redirect more pyruvate into the isobutanol biosynthetic pathway, *pflB* that encodes pyruvate formate-lyase and *ldhA* that encodes lactate dehydrogenase, which are responsible for the generation of formate and lactate, separately, were deleted in turn from BW25113.

In wild *E. coli*, *xylA* encoding xylose isomerase and *xylB* encoding xylulokinase were mainly responsible for xylose assimilation. However, one mole of ATP was needed for generating one mole of xylulose from the xylose catalyzed by *xylB*. In contrast, *E. coli* K-12 series strains including BW25113 can naturally utilize D-xylonic acid with an analog of the Dahms pathway, but lack xylose dehydrogenase activity [23]. Accordingly, the *xylD*(Cc) gene from *C. crescentus,* encoding xylose dehydrogenase, was introduced into the *E. coli* strain to construct a complete Dahms pathway from xylose to pyruvate. In the meantime, *xylA* and *xylB* were inactivated simultaneously to direct the most xylose into the Dahms pathway. The recombinant strain of BW25113 (Δ*ldhA*Δ*pflB*Δ*xylAB*) was referred to as BWL1. Next, *yagF* encoding xylonate dehydratase and *yagE* encoding 2-dehydro-3-deoxy-D-pentonate aldolase were also overexpressed in pLL1 to strengthen the carbon flux of the Dahms pathway. As a result, the biosynthetic pathway from xylose to pyruvate was obtained. 

In the Dahms pathway, two intermediates, pyruvate and glycolaldehyde, are generated from 2-dehydro-3-deoxy-D-xylonic acid using YagE. To facilitate the metabolism of glycolaldehyde, *aldA* encoding lactaldehyde dehydrogenase and *aceAK* encoding isocitrate lyase-isocitrate dehydrogenase kinase/phosphatase were also overexpressed in pLL1. 

In previous studies, an effective isobutanol biosynthetic pathway was designed by combining a branched-chain amino acid synthetic pathway from glucose and an Ehrlich pathway, with 2-keto-isovalerate serving as a precursor [24]. Similarly, to improve the production efficiency of isobutanol from pyruvate, *adhA*(Ll) encoding alcohol dehydrogenase from *L. lactis*, *kivD*(Ll) encoding 2-keto acid decarboxylase from *L. lactis*, *ilvC* encoding ketol-acid reductoisomerase, *ilvD* encoding dihydroxy-acid dehydratase, and *alaS*(Bs) encoding acetolactate synthase from *B. subtilis* were all overexpressed in pLL2. By co-transforming pLL1 and pLL2 into BWL1, a base strain of BWL2 for isobutanol production from xylose was obtained. 

**Figure 2:**
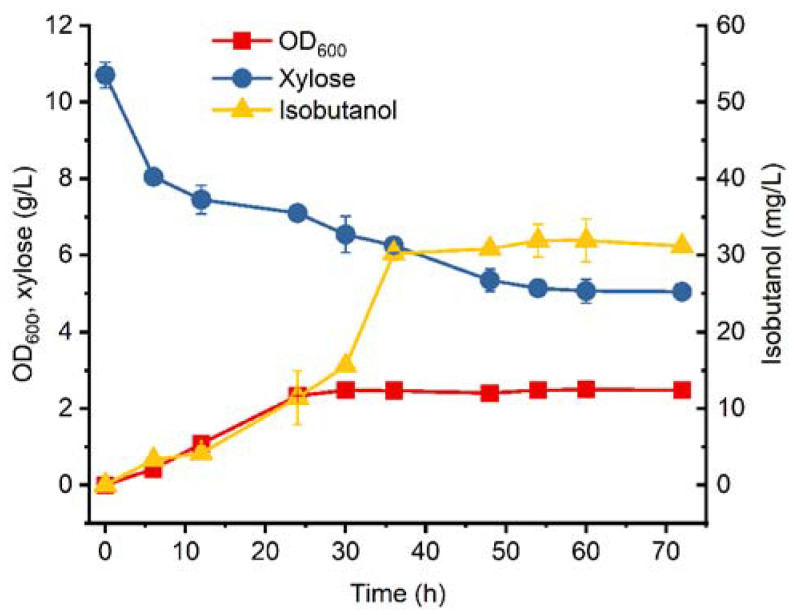
Batch fermentation of BWL2. The error bars represent standard deviations from three replicate fermentations.

To investigate the effect of the engineering strategies mentioned above, batch fermentation was performed for BWL2. As shown in Figure 2, BWL2 exhibited a relatively quick growth before 30 h, but the maximum OD_600_ was only 2.49, which was significantly lower than other isobutanol-producing *E. coli* using glucose as the sole carbon source [8]. Consistent with the growth curve, the xylose assimilation was also poor for BWL2. After 72 h of batch fermentation, 5.05 g/L of xylose remained, indicating that only 52.8% of the xylose was consumed. Compared with other reported *E. coli* strains using xylose as a carbon source for microbial production, the xylose consumption capacity of BWL2 was obviously lacking [25,26]. Nevertheless, BWL2 could produce 63.79 mg/L of isobutanol after 60 h of batch fermentation, indicating the isobutanol biosynthetic pathway from xylose worked well. To our knowledge, this is the first report about isobutanol production using xylose as the sole carbon source.

### 3.2. Engineering of Xylose Transport and Electron Transport Chain (ETC) Complex to Increase Xylose Assimilation and Isobutanol Production

In *E. coli*, there are two different xylose transport systems [27]. XylE belongs to the major facilitator superfamily of transporters [28] and is a low-affinity xylose transporter with a Km for xylose between 63 and 169 μM [29]. In contrast, XylF, XylG and XylH constitute high-affinity xylose transporters belonging to the ATP binding cassette family of transporters with a Km between 0.2 and 4 μM [29]. Accordingly, integrating another copy of *xylFGH* operon may be beneficial to the xylose utilization of BWL1.

The ETC complex is mainly responsible for bacterial respiration under aerobic conditions. Depending on ETC, the proton motive force related to ATP synthesis can be generated by the electron flow derived from the oxidation of electron donors and the reduction of electron acceptors. The ETC of *E. coli* includes two protein complexes, NADH dehydrogenase and terminal oxidase [30]. The engineering of ETC may regulate the redox state of *E. coli*, while a highly reduced intracellular state is advantageous for isobutanol production. Accordingly, ETC could be selected as a novel manipulation target for isobutanol and other reductive products [31]. 

Three ETC components, *cyo* encoding cytochrome O, *nuo* encoding NADH-quinone oxidoreductase, and *cbdAB* encoding terminal oxidases, were deleted in turn from BWL1. In addition, additional copies of *xylFGH* and *xdh*(Cc) were prepared to integrate into BWL1. To facilitate the construction process, *cyo* and *nuo* were directly replaced by *xylFGH* and *xdh*(Cc), respectively, and the resulting strain BWL3 was obtained. By transforming plasmids pLL1 and pLL2 into BWL3, the recombinant strain of BWL4 was generated.

**Figure 3:**
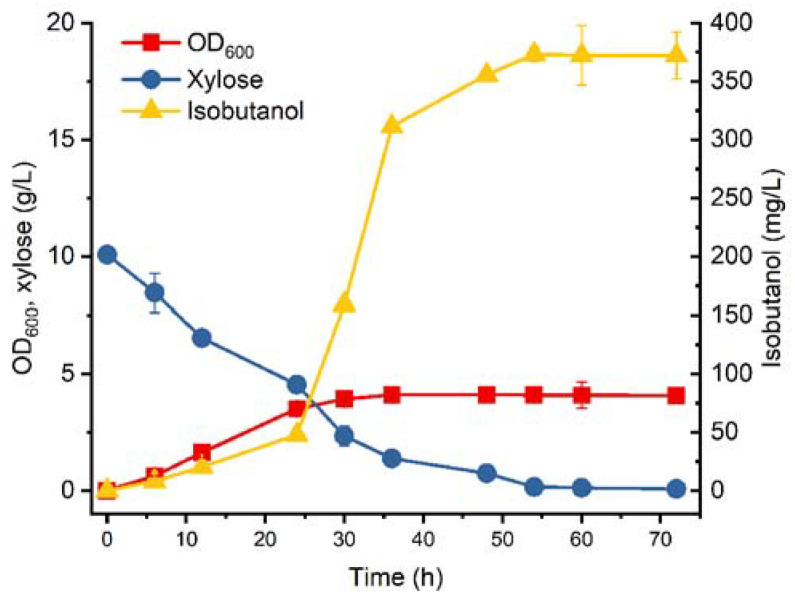
Batch fermentation of BWL4. The error bars represent standard deviations from three replicate fermentations.

Batch fermentation was then carried out for BWL4. As shown in Figure 3, after engineering the xylose transport and ETC, BWL4 exhibited improved growth with a maximum OD_600_ of 4.10, which was 64.7% higher than that of BWL2. In addition, the xylose utilization rate of BWL4 increased, and 10 g/L of xylose was nearly completely consumed after 54 h of cultivation. Consistent with our expectations, the isobutanol titer of BWL4 increased to 373.13 mg/L, representing an improvement of 484.93% that of BWL2. However, the isobutanol production titer was still relatively low compared to the recombinant strain using glucose as the carbon source [5,32]. 

### 3.3. Optimization of Xylose Supplement Concentration

**Figure 4:**
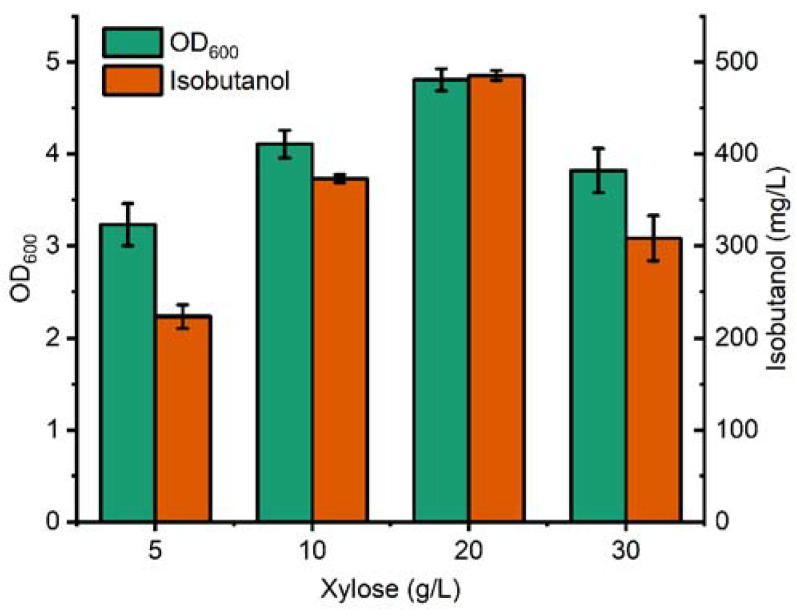
Optimization of xylose supplement concentration for BWL4 in batch fermentation. The error bars represent standard deviations from three replicate fermentations.

To further enhance the isobutanol titer, the effect of the xylose supplement concentration was investigated. Four concentration gradients, 5 g/L, 10 g/L, 20 g/L, and 30 g/L, were applied for batch fermentation. As shown in Figure 4, with the xylose concentration increase from 5 g/L to 20 g/L, the maximum OD_600_ of BWL4 was increased from 3.23 to 4.81. In accordance with strain biomass accumulation, the isobutanol production was also improved from 223.21 mg/L to 485.35 mg/L. However, the strain growth and isobutanol production were both decreased when xylose was at 30 g/L, indicating 20 g/L may be an appropriate supplement concentration for isobutanol production of BWL4. 

### 3.4. Construction of Recombinant E. coli for Isobutanol Production from Glucose–Xylose Mixture

As glucose is a preferred carbon source compared to xylose for *E. coli*, glucose was then applied as a supplemental carbon source for isobutanol production in the *E. coli*. Apart from the glycolytic pathway, three other carbohydrate metabolism pathways exist in nature, the pentose phosphate pathway, Entner–Doudoroff (ED) pathway, and phosphoketolase pathway [24]. Among them, only four enzymes are needed to obtain pyruvate from glucose in the ED pathway, which is far fewer than in the glycolytic pathway. In addition, no carbon was lost through carbon dioxide in the ED pathway [32]. Accordingly, the ED pathway was selected for assimilating glucose and generating pyruvate. To improve the performance of recombinant *E. coli* for isobutanol production using the glucose–xylose mixture as a carbon source, BWL0 was selected as the base strain for further careful engineering. When glucose and xylose both existed within the medium, assimilation of xylose occurred only when glucose uptake finished due to carbon catabolite repression (CCR). To relieve CCR and achieve a simultaneous consumption of glucose and other sugars, the deletion of PTS-related genes, such as *ptsG* encoding glucose-specific PTS enzyme IIBC components, was often carried out [33,34]. 

**Figure 5:**
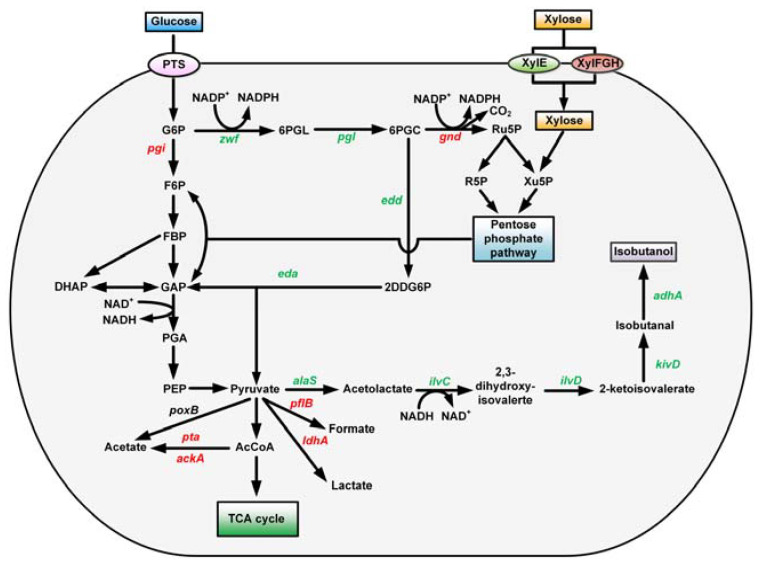
Isobutanol synthetic pathway from glucose and xylose in *E. coli*. The red color indicates genes that were deleted. The green color indicates genes directly overexpressed in plasmids.

**Figure 6:**
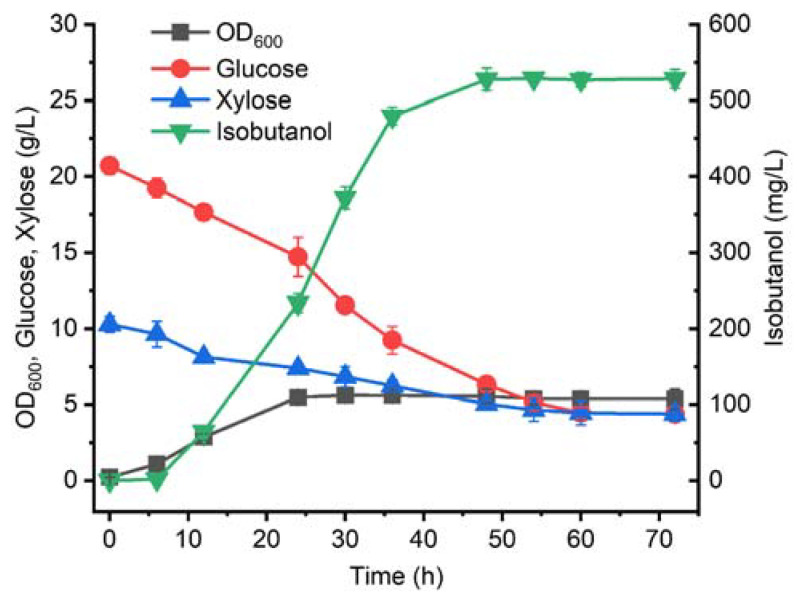
Batch fermentation of BWL9. The error bars represent standard deviations from three replicate fermentations.

As a result, *ptsG* was deleted from BWL0. Subsequently, *pgi* encoding glucose-6-phosphate isomerase was replaced with *zwf* encoding glucose-6-phosphate 1-dehydrogenase. This inactivation of *pgi* and overexpression of *zwf* could block the glycolysis pathway and redirect a carbon source into the ED pathway. Following this, *gnd* encoding 6-phosphogluconate dehydrogenase, *pta* encoding phosphate acetyltransferase, and *ackA* encoding acetate kinase were replaced by *pgl* encoding 6-phosphogluconolactonase, *edd* encoding phosphogluconate dehydratase, and *eda* encoding 2-dehydro-3-deoxyphosphogluconate aldolase, respectively, in BWL6. The inactivation of *gnd* could direct 6-phospho-d-gluconate into 2-dehydro-3-deoxy-d-gluconate 6-phosphate and block the generation of d-ribulose 5-phosphate. In addition, *pta* and *ackA* deletion could decrease the secretion of acetate [35,36]. To increase the carbon flow of the ED pathway, the intracellular expression levels of *pgl*, *edd,* and *eda* were improved. To decrease the potential metabolic burden generated by plasmids, these three genes were directly integrated into the loci of *gnd*, *pta,* and *ackA*, respectively. As a result, BWL9 was obtained (Figure 5). In order to determine the strain performance of BWL9 for isobutanol production from the 20 g/L glucose and 10 g/L xylose mixture, batch fermentation was carried out for this strain. As shown in Figure 6, after the inactivation of *ptsG*, BWL9 could assimilate glucose and xylose simultaneously. After 72 h of batch fermentation, 4.38 g/L of glucose remained, representing 21.15% of the initial glucose. In addition, only half of the xylose was consumed, which was similar to BWL2. However, BWL9 exhibited better growth than BWL2, indicated by the maximum OD_600_ of 5.39 vs. 2.49. BWL9 could produce 528.72 mg/L of isobutanol at 54 h, which was 8.3-fold that of BWL2. BWL9 also showed a slightly higher isobutanol production than BWL4 (528.72 vs. 485.35 mg/L). In addition, BWL9 was employed for isobutanol production with 20 g/L glucose as a sole carbon source. As shown in Figure S1, BWL9 exhibited a similar growth curve and glucose consumption to that of BWL9 using a glucose–xylose mixture. After 72 h of batch fermentation, 473.25 mg/L of isobutanol was achieved for BWL9, which was 11.72% lower than that of BWL9 using a glucose–xylose mixture. Perhaps the supplement of xylose was beneficial for increasing carbon flux in the pentose phosphate pathway, which could provide more NADPH for isobutanol synthesis. 

We noted glucose assimilation was impaired for BWL9 after the inactivation of *ptsG*, indicated by glucose not consumed after 72 h of cultivation. In contrast, 20 g/L of glucose could be consumed for wild MG1655 only after 24 h of batch cultivation [37]. This phenomenon was also reported by another group [38]. To improve the glucose assimilation rate and further increase isobutanol titer, additional overexpression of glucose kinase (*glk*) and glucose facilitator protein (*glf*) from *Zymomonas mobilis* may be helpful [39]. In addition, the utilization of xylose in BWL9 was unsatisfactory, as indicated by half the amount of xylose remaining in the media. The engineering of the *xylR* encoding xylose regulator has been employed to improve xylose consumption for the microbial production of chemical compounds [40]. XylR is involved in the regulation of the pentose phosphate pathway, and the overexpression of *xylR* was beneficial for xylose assimilation through the pentose phosphate pathway. In addition, manipulating redox homeostasis is crucial for isobutanol production, which can also be achieved via the engineering of XylR [41].

## 4. Conclusions

In this work, an isobutanol synthetic pathway from xylose was constructed in *E. coli* MG1655 through the combination of an Ehrlich pathway and a Dahms pathway. Engineering xylose transport and electron transport chain complex further improved xylose assimilation and isobutanol production. By optimizing xylose supplement concentration, the final strain of BWL4 could produce 485.35 mg/L of isobutanol from 20 g/L of xylose. This represents the first report about isobutanol production using xylose as a sole carbon source in *E. coli*. However, the isobutanol production titer and yield were still lower than those using glucose. Accordingly, further improvement of BWL4 is needed. First, there needs to be an optimization of the expression levels of key genes in the isobutanol biosynthetic pathway from xylose. As the whole pathway is very long, balancing isobutanol production and strain biomass accumulation is important. Perhaps an analysis of the metabolic flux can facilitate an investigation of bottlenecks in the pathway. Second, a further increase in the xylose assimilation of BWL4 is also vital for biomass accumulation and the isobutanol production titer. Apart from rational engineering, adaptive laboratory evolution (ALE) may be an effective strategy. ALE has already been applied in improving xylose assimilation of *Corynebacterium glutamicum* [42], *Saccharomyces cerevisiae* [43], and *Azotobacter vinelandii* [44]. We expect ALE will also be effective in improving xylose utilization of *E. coli*.

Lignocellulosic biomass is a renewable feedstock and is naturally available in abundance in microbial production [45]. Apart from the pretreatment and enzymatic saccharification, an efficient simultaneous utilization of mixed sugars in lignocellulosic hydrolysates, such as glucose and xylose, is also vital for low-cost biorefining [46]. We additionally investigated the isobutanol production from a glucose–xylose mixture, and achieved a titer of 528.72 mg/L from 20 g/L and 10 g/L of xylose. Further engineering of glucose kinase (*glk*), glucose facilitator protein (*glf*), and xylose regulator (*xylR*) in BWL9 may be advantageous for increasing isobutanol production to a greater extent from a glucose–xylose mixture.

## Figures and Tables

**Table 1:** *E. coli* strains used in this study.

Name	Relevant Genotype	Reference
DH5α	*F^−^, endA1, hsdR17* (*r_K_^−^, m_K_^+^*)*, supE44, thi-l, λ^−^, recA1, gyrA96,* Δ*lacU169* (*Φ80lacZ* Δ*M15*)	Lab stock
BW25113	*F^−^*, *λ^−^*, *rph-1*	Lab stock
BWL0	BW25113 (Δ*ldhA*Δ*pflB*)	This study
BWL1	BWL0 (Δ*xylAB*)	This study
BWL2	BWL1/pLL-1/pLL-2	This study
BWL3	BWL1 (Δ*cbd*Δ*xdh*::*cyo*Δ*xylFGH*::*nuo*)	This study
BWL4	BWL3/pLL-1/pLL-2	This study
BWL5	BWL0 (Δ*ptsG*)	This study
BWL6	BWL5 (Δ*zwf*::*pgi*)	This study
BWL7	BWL6 (Δ*pgl*::*gnd*)	This study
BWL8	BWL7 (Δ*edd*::*pta*)	This study
BWL9	BWL8 (Δ*eda*::*ackA*)	This study

**Table 2:** Plasmids used in this study.

Name	Relevant Genotype	Reference
pKD3	*bla*, FRT-*cat*-FRT	[19]
pKD4	*bla*, FRT-*kan*-FRT	[19]
pCP20	*bla* and *cat*, helper plasmid	[20]
pTKRed	Spc^R^, IPTG induced λRed enzymes	[21]
pCL1920	Spc^R^	[22]
pTrc99a	*bla*	Lab stock
pLL1	pTrc99a-*xdh-yagF*-*yagE*-*aldA*-*aceAK*	This study
pLL2	pCL1920-*adhA-kivD-alaS-ilvD-ilvC*	This study

## Data Availability

The data supporting this study’s findings are available upon request to the corresponding author.

## Supplementary Materials

The following supporting information can be downloaded at: https://www.mdpi.com/article/10.3390/microorganisms11102573/s1, Figure S1: Batch fermentation of BWL9 with 20 g/L glucose as sole carbon source; Table S1: Primers used in this study.
