# Supplementary material for: Engineering Escherichia coli for Isobutanol Production from Xylose or Glucose–Xylose Mixture
Source: Microorganisms. 2023 Oct 16;11(10):2573. doi: 10.3390/microorganisms11102573 (PMC10609591; doi:10.3390/microorganisms11102573)
Supplement: Supplementary file 1 [file microorganisms-11-02573-s001.zip › microorganisms-2646475-supplementary.pdf]

Supplementary materials

Engineering *Escherichia coli* for Isobutanol Production from Xylose or Glucose–Xylose Mixture

Pengfei Gu [1,*], Fangfang Li [2] and Zhaosong Huang [1]

[1] School of Biological Science and Technology, University of Jinan, Jinan 250022, China; bio_huangzs@ujn.edu.cn
[2] Yantai Food and Drug Control and Test Center, Yantai 264003, China; loveecoli@163.com
* Correspondence: bio_gupf@ujn.edu.cn

Table S1. Primers used in this study

Primers	Sequences (5′-3′)
xylAB-QF	ATTACGACATCATCCATCACCCGCGGCATTACCTGATTATGGAGT TCAATGTGTAGGCTGGAGCTGCTTC
xylAB-QR	AGATATATAGATGTGAATTATCCCCCACCCGGTCAGGCAGGGGAT AACGTATGGGAATTAGCCATGGTCC
xylAB-JF	TTACCCAGTTTCATCATTCCA
xylAB-JR	CATAGCCCAACCAGATAAACC
pflB-QF	TGTCGAAGTACGCAGTAAATAAAAAATCCACTTAAGAAGGTAG GTGTTACGTGTAGGCTGGAGCTGCTTC
pflB-QR	GTGGAGCCTTTATTGTACGCTTTTTACTGTACGATTTCAGTCAAA TCTAAATGGGAATTAGCCATGGTCC
pflB-JF	TGAGCACAGTATCGCAAACAT
pflB-JR	ATTGCGGTGTTTCTCCAGATGTG
ldhA-QF	GTAGCTTAAATGTGATTCAACATCACTGGAGAAAGTCTTGTGTA GGCTGGAGCTGCTTC
ldhA-QR	ATTGGGGATTATCTGAATCAGCTCCCCTGGAATGCAGGGGAGCG GCAAGAATGGGAATTAGCCATGGTCC
ldhA-JF	ACAGCCCGAGCGTCATCAGCAGCGTCAA
ldhA-JR	ATGGTGTTATCGAGTTCATTAAGCTGCGGG
cbd-QF	AACATTGCGCTTCATCTGGTGGCTGCTGATTGGCGTGATCCTGG TGGTCTGTGTAGGCTGGAGCTGCTTC
cbd-QR	CCAGAGAGTGTAGAGCAACACAATGGGCAAAAATATCAGCACG ATTACCAATGGGAATTAGCCATGGTCC
cbd-JF	AAATGGCGCTCTGGAGTTTG
cbd-JR	CACCACACTTTCCAGCTTCA
cyo-cmr-NF	GCTGGCATTATTTTGGGAGGTCTGGCGCTCGTTGGCCTGATCAC TTACTTGTGTAGGCTGGAGCTGCTTC
cmr-NR	CTCTAGTAGCTAGCATAATACCTAGGACTGAGCTAGCTGTAAAAT GGGAATTAGCCATGGTCC
cmr-xdh-NF	TCAGTCCTAGGTATTATGCTAGCTACTAGAGAAGGAGATATACAT ATGTCCTCAGCCATCTATCCCAGCC
xdh-cyo-NR	GCATCGCAGAATGGAACACGATGCGGCCCTGATACATGGTGAAC AGCCAGTCAACGCCAGCCGGCGTCGATCCAG
cyo-cmr-NF plus	TATCACTTGATGCAGTCCCGTTCCATGAACCTATCGTCATGGTTA CGATCGCTGGCATTATTTTGGGAGG
xdh-cyo-NR plus	GCCAGTCATCCCGCCCACCGAGAAGGTGACGATAAAACCGATG GTCCACAGCATCGCAGAATGGAACACG
cyo-JF	CAATGACGATACCTGCACCG
cyo-JR	ACACGAAGGTATGGAAGGCA
nuo-cmr-NF	GTGCTGGGTATTCCGGCAAGCGACGTCGAAGGTGTGGCAACGT TCTACAGGTGTAGGCTGGAGCTGCTTC
cmr-xylFGH-NF	TCAGTCCTAGGTATTATGCTAGCTACTAGAGAAGGAGATATACAT

	ATGAAAATAAAGAACATTCTACTCA
xylFGH-nuo-NR	CAAACGGCACTTGCGAACGGTGCGCAGTCGGCTGGTTGTTACC TTCCATCTCAAGAACGGCGTTTGGTTG
nuo-cmr-NF plus	AGAAGCAGCGTGGCTGGGTGCCGGATGGTGCGATCCACGCGAT CGCCGATGTGCTGGGTATTCCGGCAAGCGACGTCGA
xylFGH-nuo plus	GTCCTGGAATTTGTTCCACGCCTGCGGGGAGTTCCAGCCCGGCG CCCAGGCAAACGGCACTTGCGAACGGTGCGCAGTC
Nuo-JF	GTCAGTCCTTCGGCGATTTC
Nuo-JR	CCACAAACCGAGGCTTTTGA
ptsG-QF	CACGCGTGAGAACGTAAAAAAAGCACCCATACTCAGGAGCACT CTCAATTGTGTAGGCTGGAGCTGCTTC
ptsG-QR	GTAAAAAAGGCAGCCATCTGGCTGCCTTAGTCTCCCCAACGTCT TACGGAATGGGAATTAGCCATGGTCC
ptsG-JF	TCGCCCGTCTGTTTCACATCG
ptsG-JR	AGAGTTGACGGTCGGCGGTGGT
pgi-cmr-NF	CGCTACAATCTTCCAAAGTCACAATTCTCAAAATCAGAAGAGTA TTGCTAGTGTAGGCTGGAGCTGCTTC
cmr-NR	CTCTAGTAGCTAGCATAATACCTAGGACTGAGCTAGCTGTAAAAT GGGAATTAGCCATGGTCC
cmr-zwf-NF	TCAGTCCTAGGTATTATGCTAGCTACTAGAGAAGGAGATATACAT ATGGCGGTAACGCAAACAGCCCAGG
zwf-pgi-NR	GTTGCCGGATGCGGCGTGAACGCCTTATCCGGCCTACATATCGA CGATGATTACTCAAACTCATTCCAGGAACGA
pgi-cmr-NF plus	CTCAACATTACGCTAACGGCACTAAAACCATCACATTTTTCTGTG ACTGGCGCTACAATCTTCCAAAGTC
pgi-cmr-NR plus	GCATCGACCTGTAGGCCTGATAAGACGCGACCGCGTCGCATCAG GCATCGGTTGCCGGATGCGGCGTGAA
pgi-JF	TAGGATTAACCTCACGGTATG
pgi-JR	ATCACAGGACTGGCTCCTCCAA
gnd-cmr-NF	TCCAGGCCGCGAGCATTCAGCGCGGTGATCACACCTGACAGGA GTATGTAGTGTAGGCTGGAGCTGCTTC
cmr-NR	CTCTAGTAGCTAGCATAATACCTAGGACTGAGCTAGCTGTAAAAT GGGAATTAGCCATGGTCC
cmr-pgl-NF	TCAGTCCTAGGTATTATGCTAGCTACTAGAGAAGGAGATATACAT ATGAAAATCACCATTTCCGGTACTG
pgl-gnd-NR	GCCCGGTGCAATATACGCCGGGCCTCAATTTTATTGTTGGTTAAA TCAGATTAGTCGCTGCCAAAGAGAT
gnd-cmr-NF plus	ATAAGCTATTTATACTTTAATAAGTACTTTGTATACTTATTTGCGA ACATTCCAGGCCGCGAGCATTCAG
pgl-gnd-NR plus	GTTAATATTAAGCAGTGAAATTTAGTCTATAAGATATTTGGCAAA AAAAAGCCCGGTGCAATATACGCCG
gnd-JF	TGGCTGGTGTCAGACTTCATT
gnd-JR	GCGATTAGAAGCCCGTTTGAC
pta-cmr-NF	GGTGCTGTTTTGTAACCCGCCAAATCGGCGGTAACGAAAGAGG

	ATAAACCGTGTAGGCTGGAGCTGCTTC
cmr-edd-NF	TCAGTCCTAGGTATTATGCTAGCTACTAGAGAAGGAGATATACAT ATGAATCCACAATTGTTACGCGTAA
edd-pta-NR	TTATTTCCGGTTCAGATATCCGCAGCGCAAAGCTGCGGATGATG ACGAGATTAAAAAGTGATACAGGTTG
pta-cmr-NF plus	AAGACGCGAGCCGCCTGACTGCCTGATTTCACACCGCCAGCTC AGCTGGCGGTGCTGTTTTGTAACCCGC
edd-pta-NR plus	ATCAGAAAGGATTAATGCAAATTAAGAGAATAAAAAACCGGAA ATAGTGATTATTTCCGGTTCAGATATC
pta-JF	CAATGGACGTTTACTGCCACC
pta-JR	CCAGGTGGCAAGGCTGGTTAA
ackA-cmr-NF	CTATGGCTCCCTGACGTTTTTTTAGCCACGTATCAATTATAGGTAC TTCCGTGTAGGCTGGAGCTGCTTC
cmr-eda-NF	TCAGTCCTAGGTATTATGCTAGCTACTAGAGAAGGAGATATACAT ATGAAAAACTGGAAAACAAGTGCAG
eda-ackA-NR	GATTTGGCGGGTTACAAAACAGCACCGCCAGCTGAGCTGGCGG TGTGAAATTACAGCTTAGCGCCTTCTA
ackA-cmr-NF plus	TGCAGTGCATGATGTTAATCATAAATGTCGGTGTCATCATGCGCT ACGCTCTATGGCTCCCTGACGTTTT
eda-ackA-NR plus	GGGATCAGCATAATAATACGGGACACGGTTTATCCTCTTTCGTTA CCGCCGATTTGGCGGGTTACAAAAC
ackA-JF	CGATTATCCGGCGTTGACATG
ackA-JR	CGCACGCACGATAGTCGTAGTCTGAT

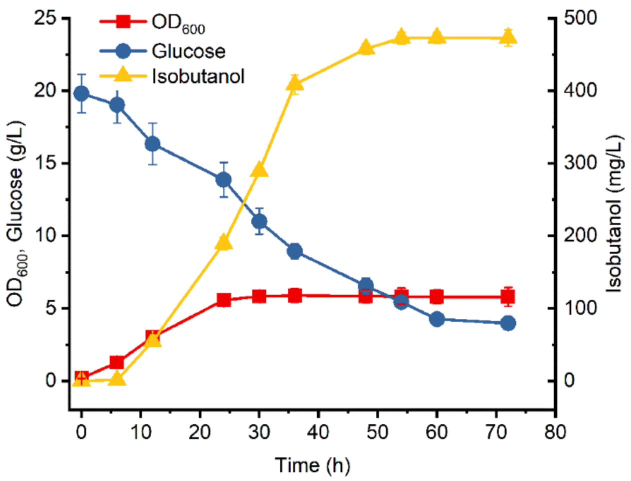

Figure S1. Batch fermentation of BWL9 with 20 g/L glucose as sole carbon source. The error bars represent standard deviations from three replicate fermentations.
